# ‘But you don’t look sick’: a qualitative analysis of the LUPUS UK online forum

**DOI:** 10.1007/s00296-020-04726-x

**Published:** 2020-10-26

**Authors:** Melanie Sloan, Michael Bosley, Moira Blane, Lynn Holloway, Colette Barrere, David D’Cruz, Chanpreet Walia, Felix Naughton, Paul Howard, Stephen Sutton, Caroline Gordon

**Affiliations:** 1 Behavioural Science Group, Institute of Public Health, University of Cambridge, Forvie Site, Robinson Way, Cambridge, CB2 0SR UK; 2 Patient and Public Involvement in Lupus Research Group, Institute of Public Health, University of Cambridge, Cambridge, UK; 3 The Louise Coote Lupus Unit, Guy’s and St Thomas’, NHS Foundation Trust, London, UK; 4 LUPUS UK, St James House, Romford, Essex UK; 5 Behavioural and Implementation Science Group, School of Health Sciences, University of East Anglia, Norwich, UK; 6 Rheumatology Research Group, Institute of Inflammation and Ageing, College of Medical and Dental Sciences, University of Birmingham, Birmingham, UK

**Keywords:** Lupus, Patient views, Patient–physician interaction, Quality of life, Misdiagnosis, Holistic care

## Abstract

Lupus is a multi-system autoimmune rheumatic disease with increased morbidity and mortality. Some manifestations are life-threatening with many aspects of living with the disease, difficulties in diagnosis and accessing appropriate medical care, having an impact on quality of life. The disease itself, and these patients’ perspectives, are currently poorly understood and under-researched. The LUPUS UK forum of conversations between over 25,000 members provides a rich environment to explore the views of these patients. Conversations on the LUPUS UK online forum were qualitatively explored using virtual ethnography and thematic analysis. The forum itself and positive medical relationships were widely considered to provide a means of support, understanding and validation. Forum members expressed difficulties in diagnosis, disease management, and the psychological and physical impact of living with an unpredictable, poorly understood disease, often with life-changing symptoms. Invalidating personal, social and medical environments were perceived as exacerbating these difficulties. Delays in diagnosis and misdiagnoses were frequently discussed as causing significant damage, especially when symptoms were disbelieved or dismissed. Invalidation was the key theme with further themes of: Uncertainty, Medical (mis)communications and misunderstandings, Navigating health systems and Resilience and support*.* Although effective care and support was reported by some members, the negative impact of living with an incurable, life-changing disease was often exacerbated by perceived invalidation, uncertainty, and difficulties in multiple areas of members’ lives. Improved knowledge of the disease and greater support at all stages of the diagnostic journey could improve outcomes and quality of life for these patients.

**Electronic supplementary material:**

The online version of this article (10.1007/s00296-020-04726-x) contains supplementary material, which is available to authorized users.

## Introduction

Systemic lupus erythematosus (SLE) is a chronic multi-system autoimmune rheumatic disease [1] causing significant morbidity and mortality [2, 3]. In addition, there is often a severe impact on quality of life (QoL) [4, 5]. This includes: increased prevalence of multiple neuropsychiatric manifestations [6–8], employment difficulties [5, 9], and debilitating fatigue [5, 10–12]. The unpredictability, lack of understanding/recognition of the disease and diverse manifestations generate additional challenges for patients and physicians.

Lack of definitive tests means diagnosis and management largely relies on individual physician knowledge, with many studies reporting inadequate training and knowledge [13, 14]. Delays in diagnosis and misdiagnoses are common with previous studies reporting an average of 6–7 years to diagnosis [11, 12, 15], and approximately 50% of patients initially misdiagnosed [11, 12].

Our previous research has found that diagnostic difficulties and negative medical interactions can generate persisting psychological damage and insecurity, particularly amongst those who were met with disbelief or were given psychosomatic or mental health (MH) misdiagnoses [11, 16].

Despite the World Health Organisation (WHO) increasingly recognising the importance of wellbeing, and encouraging governments to provide education and tools for self-management and care in chronic diseases [17], many patients with lupus report that there is limited self-management education. They thus often rely on peers for knowledge transfer and emotional support. Peer support from those who experience the same challenges of living with the same chronic health condition [18, 19] is increasingly a focus of research.

Previous studies have identified a need to focus on QoL and holistic care [20, 21], increased awareness of the wider needs of this patient group [4, 5], and the impact of the patient–physician relationship [16, 22].

Forum analyses are increasingly used in qualitative research [23] as a cost-effective method of analysing the experiences of thousands of patients to find common themes. The LUPUS UK forum has over 25,000 members with multiple conversations and opinions of a large group of patients who are communicating openly with peers. This forum analysis will build on our previous work [11, 16] with the aim of giving these patients a wider combined voice, and identifying unmet needs, values, concerns and preferences, thus enabling patient-centred improvements to be considered.

## Methods

### Participants

Demographic information is limited as the majority of participants use pseudonyms. The site moderator, LUPUS UK, estimates that the vast majority of active members are from the UK and have SLE. The site also includes patients with related autoimmune diseases such as undifferentiated connective tissue disease (UCTD), Sjögrens, and in the diagnostic uncertainty stage. The analysis included all active members of the forum. From November 2018 to January 2019, there were an average of 2649 active (posting at least once) members per month representing 11.8% of the 22,484 members as of 6 February 2019.

### Data collection and analysis

Two methodological approaches were combined to study this population. First, virtual ethnography [24], where seven members of the research team were fully immersed in the community, either as patient members or moderators. These researchers had a total combined time in the community of over 30 years. Second, forum posts were analysed thematically in reverse chronological order from October 2019 until theoretical saturation was reached with no new concepts emerging [25] (March 2019). This involved (1) developing and agreeing a broad coding scheme, (2) coding the data, (3) combining and comparing the posts/responses with other quotes on the same theme, (4) refining and re-coding, (5) identification of commonly occurring themes [25], and (6) further review of selected relevant historical posts as far back as September 2012. Particular attention was paid to deviant findings to strengthen validity of the findings [26]. Ethical approval was obtained through the Cambridge Psychology Research Committee—PRE.2018.120. Permission to quote individuals was obtained by LUPUS UK staff. Questions to clarify that emerging themes fully reflected the members’ experiences, and later the draft paper, were posted on the forum to discuss and agree key findings with the community.

Further details of methods, ethical considerations and researcher reflexivity can be found in supplementary information 1.

To ensure that the qualitative analysis also reflected the quantities of subject matter posted, five co-authors carried out content analysis of a month of forum posts each (randomly generated from January 2016 to April 2019).

## Results

The six most frequent types of posts (as a % of total posts) were (1) asking for advice on symptoms, 18%; (2) negative medical appointments/interactions, 17%; (3) medication/test results queries, 15%; (4) general advice given/sought, 13%; (5) emotional/mental health/struggling to cope, 8%; and (6) search for diagnosis, 7%. The ratio of positive to negative posts discussing medical interactions was 1:6.5.

The overarching theme was Invalidation in multiple domains (medical, societal and self) of forum members’ lives. Further themes included: Uncertainty, Medical (mis)communication and misunderstandings, Navigating health systems and Resilience and Support.

### Invalidation: medical

In addition to ensuring appropriate support and treatment, a diagnosis was widely felt to confer personal, medical and social acceptance, yet delays and misdiagnoses were frequently reported.

While many members reported severe manifestations of the disease including organ damage, early symptoms reported on the forum were often non-specific, such as migrating pain, severe fatigue, fevers and rashes. A desire for physicians to persist in ‘joining the dots’ in pursuit of the correct diagnosis was frequently expressed. Although some achieved prompt diagnosis, the diagnostic journey was widely reported to be extremely invalidating and distressing, with words including ‘unheard’, ‘rejected’, ‘shame’, ‘guilt’, ‘dismissed’, ‘desperation’, ‘fear’, ‘abandoned’ frequently occurring, along with reports of a loss of dignity and self-worth.

Some forum posts detailed how individual physicians provided compassionate support during the years in the ‘diagnostic wilderness’. However, a more frequent type of post was when the continued search for a diagnosis was reported to have led to misdiagnoses of health anxiety/hypochondria, psychosomatic or mental health issues. Posts detailing these misdiagnoses often expressed great distress and distrust, and a perception that patients’ self-reported symptoms were not always believed or validated, including post-diagnosis.

Patient quotes of experiences of medical invalidation, including opposing validating experiences of a diagnosis and supportive physicians, are shown in Table 1.

**Table 1:** Medical invalidation/validation quotes

The importance of viewing symptoms holistically in achieving a diagnosis
The sense of blackness that would pass over me at appointments, fearing the nothing wrong statement or just ‘anxiety’. The despair of walking away with no help still feeling very ill and with no life. Just that one consultant who looks deeper and changes things for the better… When the dots are joined up it makes a big difference to quality of life. There are some great dedicated doctors out there and they really are trying very hard to help us (Female, UK, 50s)
My final diagnosis was very quick…thanks to an amazingly observant GP – but in hindsight I realised I had had lupus for years before that but because the symptoms all appeared separately nobody pieced the jigsaw together. Lupus is notoriously hard to diagnose because it affects everybody differently and there are so many different symptoms (Female, 70s)
The ‘fight’ for validation of multi-system symptoms that fluctuate over time
You’re fine and then suddenly something is wrong and you know it, but instead of a straightforward test and answer, you have to fight to be believed, then it goes this way and that from one department to another and back again and not everyone is on the same page and you have to explain everything to every consultant every time (Female, England, 50s)
‘Relief’ and validation on diagnosis
For me it took about 10 years to diagnosis…I kept thinking I was just a tired unmotivated person. It was a relief to get the diagnosis because you stop thinking you are crazy or imagining symptoms (Female, US, 60s)
The psychological benefits of validation during the—often prolonged—diagnostic uncertainty phase
[GP] was the best example of a compassionate, intelligent and very wise Doctor giving me the wonderful care and sympathy despite no definitive diagnosis. A positive Doctor gives as much psychological support as any drug (Female, UK, 60s)
Residual distress and anger towards physician(s) due to disbelief and invalidation during the diagnostic journey
Did my illness make me this unwell or did my battle to be heard make me this unwell, maybe the battle just depletes us… I just completed a self referral for counselling. I think now I have some answers it’s time I dealt with the problems this journey has caused me. I’ve got such a build up of hate and anger towards 1 doctor! (Female, England, 30s)

### Invalidation: societal

The frequent lack of medical clarity, a widespread lack of knowledge about the disease and the invisibility of many symptoms resulted in posts about limited understanding and disbelief in the reality and severity of symptoms from patient contacts and wider society, with members often being told “but you don’t look sick’’. This was reported to have caused difficulties with family, friends, work, and school, resulting in posts about relationships being severely challenged and sometimes failing.

Similar problems led to significant hardship and distress when applying for and using welfare benefits and concessions such as Blue Badges. Forum members often reported being disadvantaged compared to more visible or widely understood conditions such as cancer, rheumatoid arthritis, diabetes and hypothyroidism, in terms of treatment, medical, and social support systems.

### Invalidation: self

The consequences of the organic disease itself were considerable. Some members expressed a deep sadness and felt that they were a reduced version of their previous self. Many were unable to work, socialise, or fulfil their caring roles as they would like. Forum members, particularly those with young children, commonly felt guilt and lowered self-worth. Managing and accepting these chronic diseases, medications, limitations and building relationships with physicians was often felt to be hindered by traumatic diagnostic journeys and by having a diagnosis that remained subject to change, progression and uncertainty.

Fatigue was regularly described as extremely debilitating and isolating, predominately due to causing an inability to participate fully in life, but also due to the incomprehensibility of those who have not personally experienced it. Physicians and people without chronic diseases expressing, or implying, doubt that this pathological fatigue could be far more profound than ordinary tiredness left many members feeling inadequate and ‘lazy’.

Reductions in physical abilities were often compounded by cognitive impairment, causing difficulties in work and relationships. Changes to appearance caused by the disease itself (for example, from skin involvement) and from medications (particularly weight gain from steroids) were also discussed as distressing and highly damaging to self-esteem.

These features were often exacerbated by an internalisation of the invalidating attitudes of others. Many reported that the initial disbelief they encountered from physicians made them doubt their own judgement—even their ‘sanity’. The vast majority of forum posts following a diagnosis expressed great relief on having an explanation for their symptoms: of validation and vindication.

Table 2 includes participant quotes documenting the emotional and psychological damage from societal and self-invalidation.

**Table 2:** Societal and self-invalidation

Invalidation and loss of dignity from difficulties obtaining welfare (Personal Independence Payments –PIP) disability benefits
The whole [PIP] process is degrading and mentally painful. Why is it a matter of guilty until proven innocent I don’t know. Maybe we’ve moved beyond the ‘treated with dignity and respect’ mantra..the feeling of being thought of as a scam artist or downright liar is worse than losing the money (Female, England, 60s)
My PIP was stopped, we are barely surviving…British Gas are on my tail…motability given me a date to reclaim my car. I am tired, the way and manner the PIP rug is pulled off one is horrible. I want to work but I’m not well enough…the stress that comes with the benefits system, slowly chips your life away even before the lupus gets to you.(Female, UK, 30s)
Lack of understanding and societal support for lupus compared to more well-known diseases
I feel ashamed that I’ve thought it myself, that cancer would be better…it has such a huge lobby (researchers, charities, specialist status in law…) and most of all, everyone has empathy for someone with cancer (Female, Scotland, 50s)
Impact of the changes to appearance and reduction in quality of life for patients and their families
The weight gain, hair loss, teeth loss. On and on and on! I feel so pathetic, and ugly, and embarrassed by my appearance, I try to walk tall and act like I don’t care, but I do. It’s humiliating. The giving up all the activities I love. Missing out on so much and feeling I’m ruining my husband’s life too (Female, USA, 60s)
Over-whelming fatigue and subsequent guilt and self-invalidation
Feel like the worst parent in the world, my poor kids…I’m sat there literally dying from fatigue…. It’s like wasting life …I just feel like giving up…I feel so useless and look at the mess in the house and the kids in their pjs all day and feel like a useless mum (Female, England, 40s)
Suicidal thoughts and a loss of self-worth from lack of diagnosis and support
I’ve been in a very low place too when nobody seemed to understand what was wrong with me and worse still didn’t seem to care!! I felt worthless. I too thought of ending my life and had to get emergency counselling as well as medication for severe depression. (Female, Scotland, 60s)
Feelings of worthlessness from invalidation and loss of former identity
At what point does one's use in the world expire with this disease. So, if it takes away my love of walking, writing, thinking, functioning, being free, spending quality time with my daughter, what is left of 'me' and do I become a 'burden'…lost and worthless…my quality of life is slowly dropping away—everything that I am seems to be disappearing in this horrible set of conditions I have and the awful way we have to prod and poke the medical profession to help (Female, UK, 50s)

### Uncertainty

Living with an unpredictable multi-system disease subject to relapsing, remission, progression and evolution of symptoms caused great uncertainty and difficulties in planning and managing lives daily and long term.

A stable and unchanging diagnosis was widely considered as being important to acceptance. Uncertainty seemed especially difficult to accept by the newly diagnosed, and they were often guided by supportive posts from moderators and experienced members.

A perceived lack of consistency in diagnostic criteria and terminology—even within the same rheumatology department—created great uncertainty and insecurity among patients. There were multiple reports of a diagnosis that had been removed or changed, and life-changing medications withdrawn. This was often perceived to be due to a change of rheumatologist, or an over-focus on (often inaccurate or outdated) test results, despite symptoms remaining unchanged.

### Medical (mis)communications and misunderstandings

Members reported basic misunderstandings of the disease, both by themselves and physicians, especially in the early stages of diagnosis. Although some received clear explanations, many felt they were given limited information, with the forum frequently consulted on symptoms, test results and medications.

The contents of face-to-face communication, clinic letters and reports were frequently discussed. A failure to empathise, to acknowledge patient concerns, and to include the patient in the sharing of all results and reports were often identified as a source of frustration and disempowerment.

Miscommunication was found to often exacerbate other concerns. Labels such as UCTD and ‘incomplete’ lupus were sometimes felt to be poorly explained and not a ‘proper’ diagnosis. While reassuring to some, ‘mild’ lupus was felt to be dismissive to many who reported that, although they understood it may mean non-organ involvement, it felt undermining given the extent of their life-changing symptoms.

Many members accrued additional symptoms and autoimmune diagnoses over time and largely accepted that is the nature of autoimmunity. Some were given non-inflammatory diagnoses such as fibromyalgia, functional disorders or ME/CFS, usually as co-diagnoses which some members accepted could be an accurate representation of some symptoms. However, many strongly felt these that were ‘fobbing off’ misdiagnoses given by physicians felt to be ‘*guessing’* due to insufficient knowledge of complex autoimmunity, with little/no discussion, objective evidence, or scientific rationale.

Table 3 contains some Forum members’ quotes highlighting common issues with uncertainty and medical communications.

**Table 3:** Uncertainty, medical (mis)communications and misunderstandings

The desire for a named and acknowledged disease to explain life-changing symptoms
I just find it frustrating not to have a name for the disease that has pretty much ruined my life (Female, UK, 30s)
Difficulties from changing diagnoses
So after being told I had lupus, telling my employer and getting the sack over it, a 2nd rheumatologist said it was UCTD. No one has bothered explaining anything (Female, Wales, 30s)
Great appreciation for physicians managing diagnostic uncertainty compassionately
He went on to say sorry… it gives you no answer but lovely to meet you and I wish you well and hope you find the doctor with the answers, I’m sure it’s there it’s just knowing who to see. So still no answers but treated like a human being… could have hugged him… just plain honesty and empathy and it was a heavenly change of experience. (Female, UK, 50s)
Frequent misunderstandings of these diseases leading to diagnostic delays and misdiagnoses
(GP) said if I had lupus my kidneys would be involved (Female, Canada, 60s)
He [GP] hadn’t heard of the dsDNA test [positive]… I was prescribed antidepressants…after frequent visits he concluded ‘you’re a glass half-empty kind of person’. (Female, Scotland, 50s)
Difficulties interpreting and/or accessing test results
I don’t even know what tests each doctor has done. If I don’t ask they either don’t tell me or dazzle me with stuff I have no idea about they may as well be speaking Chinese (Female, Scotland, 50s)
If a GP cannot interpret a result, that’s their problem- they shouldn’t use that as an excuse to withhold it from me. Likewise for a consultant to withhold a result because they think I cannot be trusted with it is paternalism gone mad…I cannot tell you how much better I feel having got the blood results in my hand – even if they aren’t the best. Being denied information about your own illness is completely undermining—and wrong. (Male, Wales, 50s)
The importance to patients of compassionate, attentive communication verbally and in writing
I’ve just received my rheumy review letter and am really down and very tearful…feel hopeless and helpless suddenly. I’ve plummeted on reading this cold letter…I know we are a miniscule (and clearly forgettable) person in their lives, they have such huge caseloads… but after 3–4 months of waiting what they say and write afterwards means so very much (Female, UK, 50s)
The view that autoimmunity is widely misunderstood leading to over and misdiagnosis of non-organic syndromes
As I frequently drag my collection of body parts around multiple ‘ologies’ I do wonder when it was that I stopped being…well…a systemically connected human body. I ‘m not denying that conversion or functional disorders exist…just that these labels are often too easily plucked out of nowhere…often through lack of rigour, willingness or the time to get to the root cause of what I believe are some of the trickier symptoms of autoimmune disease (in all their ugly guises) (Female, England, 60s)
I’m increasingly incensed and hope to lobby for the word functional to be challenged until all the established and newer methods of testing for organic conditions are available to all in UK. Particularly for those of us in the devolved nations who can’t even access London testing due to not having a right to an out of Scotland/Wales second opinion (Female, Scotland, 50s)

### Navigating health systems

Although some reported that support was quickly available, there were more frequent posts detailing difficulties accessing medical support, ‘gatekeeper’ receptionists/GPs, irregular and postponed appointments and declined referrals. Patients and physicians were often perceived as being trapped in an inefficient, over-burdened system. There was a widespread sense of injustice that limited reserves of physical and mental energy were required not just to ‘fight’ the disease, but also in overcoming barriers to obtaining medical support, both pre- and post-diagnosis.

Geographical inequalities in care were regularly discussed. In particular, members in parts of Wales discussed a perception of poor local rheumatology services. These members, and others, reported great distress—and sometimes irreversible organ damage—with frequent descriptions of being refused access to SLE specialists due to ‘gate-keeper’ policies.

Personal characteristics were discussed as influencing diagnosis, especially apparent among female members perceiving that physicians may be more inclined to give psychological/MH misdiagnoses like ‘anxiety’ and ‘stress’ based on their gender and stage in life. As many became symptomatic in the child-rearing years, the stress of ‘being a new mum’, ‘having teenagers’, etc., were sometimes initially given as reasons for their symptoms, with symptoms in older females often attributed to the menopause, age-related decline and in one member ‘empty nest syndrome’. However, although males are much in the minority as SLE patients and on the forum, their conversations revealed that their diagnostic journey was often no easier.

A proportion of UK patients reported that diagnostic/care difficulties or NHS waiting times led them to seek a diagnosis from SLE specialists privately, creating diagnostic and treatment inequalities between socioeconomic groups.

Much appreciation was expressed for rheumatologists who took the holistic view of the patient and co-ordinated highly effective multi-disciplinary care. Conversely, frustration was expressed when symptoms were not seen systemically, or medical specialisms seemed to treat the patient as a collection of unconnected body parts. Some members complained that they felt passed ‘like an unwanted parcel’ around the ‘ologies’ with ‘merry go rounds’ of superficial investigations that failed to take account of the complex and difficult to detect manifestations of SLE/CTDs. Members reported being regularly discharged with ‘reassurance’, yet leaving them with debilitating and/or concerning unexplained symptoms, until tests verified the patients’ experiences. This occurred both pre- and post-diagnosis and added to the perception of invalidation and self-reported symptoms not being believed.

Table 4 contains quotes of positive and negative experiences of accessing appropriate care, including multidisciplinary care, inequalities and administrative failings.

**Table 4:** Navigating health systems

The impact of cancellations and difficulties accessing medical care
To them it’s a cancelled appointment, to me it’s my life (Female, England, Teens)
I’ve no letter and no booking for my next appointment. There are no lupus nurse appointments and no one’s answering the telephone. I know things are terrible in hospital but it doesn’t alter how horrible it feels to be abandoned…the horrible irony of it all is if we got more preventative services before we got critical our care would be a lot cheaper and we’d be more likely to be working…I just feel too tired to keep on saying the same thing over and over. (Female, UK, 50s)
Views on inequalities of care
I also live in wales and can echo the woeful lack of awareness, facilities, therapies, access to care and support! (Female, Wales, 30s)
Apart from being female, it was pretty obvious that me being relatively poor, impaired by trauma, illness, brain fog and being unable to think quickly on my feet made it near impossible to get the time required for appropriate SLE care (Female, Australia, 50s)
Experience of co-ordinated multidisciplinary care
Despite the negative bloods, I had a few years of remarkably enlightened consultants who kept each other in the loop and worked together on it (Female, UK, 40s)

### Resilience and support

Close working relationships with physicians were reported by many members, often with such an overwhelming feeling of relief and gratitude to the clinician(s) who diagnosed and/or gave them compassionate care that they were frequently described in terms of being ‘*stars’ *or* ‘heroes’* with a feeling of great security and attachment.

Honest and empathetic communication from physicians was highly valued, with many posts highlighting the importance of listening with compassion to a patient’s subjective experiences. The importance of the role that allied health professionals, such as nurses and physiotherapists, play was discussed. Members reported many positive interactions, and a perception that such professionals may have more time to focus on listening to the patient, and helping with the practical challenges of the disease.

Some members reported how diagnosis, medical support and acceptance gave them strength and allowed them to validate their own symptoms by listening to their bodies and pacing activities. Many members also detailed support from family members and friends, both practical and emotional. Although lives were clearly substantially altered by the disease, there were regular inspiring posts by members who were finding a sense of purpose and achievement by adapting activities around the limitations of the disease.

The forum itself tended to be discussed in extremely positive terms. It performs several complementary functions, including facilitating friendships and reducing isolation and loneliness, with active moderators ensuring a safe and respectful environment. Members shared symptoms and problems and exchanged knowledge, research papers and tips on managing both the disease and medical encounters. This often led to improved medical confidence and medical relationships.

Table 5 contains forum member quotes highlighting the great value placed on empathetic physicians and the LUPUS UK forum in providing compassionate support.

**Table 5:** Resilience and support

Supportive medical relationships
Mine (‘star GP’) once phoned me at 7 pm on a Friday night to check I was OK! …he always books me in for his last appointment as he doesn’t want me to feel rushed (Female, Scotland, 40s)
My wonderful consultant watched as I cried in her office [on dx with SLE]. I asked why she believed me when I’d been dismissed as an awkward patient ‘because when a patient tells me something I choose to believe them’ (Female, England, 30s)
I feel I have had the best of treatment and have been shown understanding, compassion and also been involved in the development of my treatment plan. (Female, UK, 70s)
Learning to adapt to the limitations of the disease, with compassionate guidance from clinicians
I went to get some physio and she was so positive ‘we will get you back into shape’ and she was a great listener…. I thought I really need encouragement right now and positive speak (Female, Wales, 50s)
I was very tearful…she [lupus nurse] was very kind…held my hands and said all of this has unsettled your Sjögrens, no wonder you feel so low. Walking out of there I already felt a little uplifted. Nurse did not mention antidepressants, give an eye roll, look at her watch…she gave compassion, understanding and reassurance. (Female, UK, 60s)
The value of peer support and understanding obtained from the forum
We all hit that wall of despair, but if we let each other know, as you all always do, that there are those who care, who can lift us for the moment so we can shake it off and keep going, I think it helps see us through…I can’t tell you how many times I’ve read posts and have cried, been so angered, or laughed, and felt inspired by all of you! (Female, 60s, US)

**Fig. 1:**
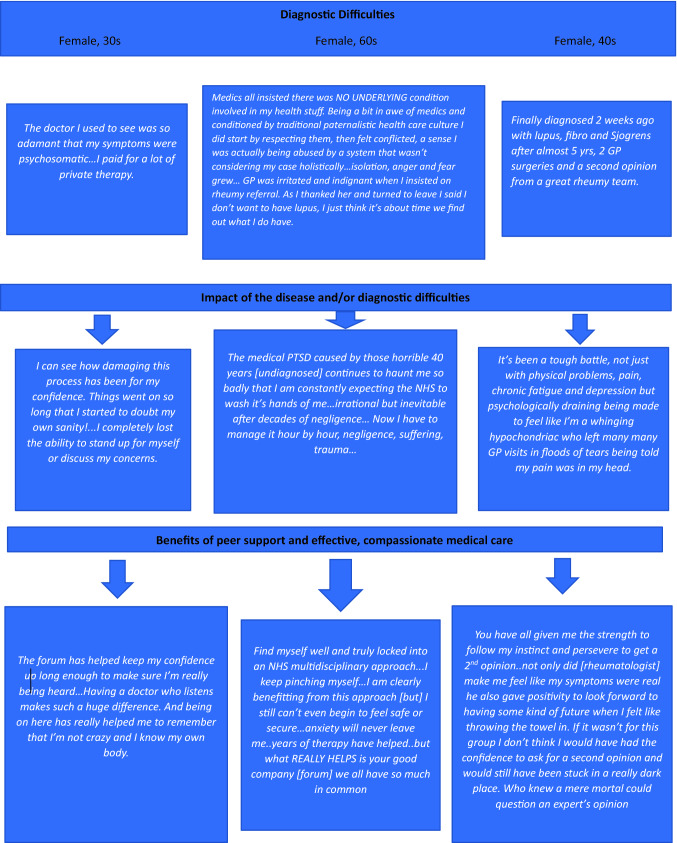
Examples of three typical diagnostic journeys

Figure 1 gives examples of three forum members’ diagnostic journeys, highlighting the damage caused by delayed diagnosis and ‘disbelieving’ physicians, and the redeeming impact of supportive physicians and peers.

## Discussion

A feeling of relief was usually described as the overwhelming emotion on diagnosis. Considering SLE is often a life-changing, sometimes life-threatening, incurable disease with many unpleasant manifestations, this is a strong indication of the degree of trauma often suffered on the journey to diagnosis and the desperation for medical help and validation. This research has identified that many forum members experienced a distressing combination of life-changing symptoms with often traumatic diagnostic journeys and a perception of invalidation in multiple areas of their lives.

Initial symptoms were often reported on the forum as appearing sequentially and insidiously, with patient and physician encountering difficulty in identifying an underlying disease process frequently leading to misdiagnoses. Those in the ‘not yet correctly diagnosed’ stage are clearly a highly vulnerable, often medically neglected group, at risk of potentially irreversible damage to their physical and mental health.

SLE is often reputed to be an ‘invisible’ disease with both social and medical diagnoses seemingly reliant upon visual indicators of disease, with an often prolonged period of time before symptoms are validated by a diagnosis [27]. Validation is a key theme identified in both the existing literature and this research. Santiago et al. found that invalidation in all rheumatic diseases was associated with psychological factors, loneliness and greater pain, and requiring intervention [28]. Our research has added greater depth to previous research findings, that the lack of perceived consideration for subjective symptoms leaves many patients feeling disbelieved and dismissed [11, 16, 29, 30] and remains an ongoing issue. Price and Walker found that for some, the SLE diagnostic journey can reflect the nature of the disease in terms of uncertainty and ‘chaos’ [31]. Our study is in agreement, and has provided additional evidence, to findings of studies of other rare diseases that the search for diagnosis can be very distressing and finally obtaining a diagnosis can be extremely validating [32].

In agreement with our previous research [11, 16], we found that mental health/psychosomatic misdiagnoses were perceived as particularly damaging, resulting in posts demonstrating insecurity and fear of physician disbelief even in subsequent positive medical relationships. Many forum participants reported that they eventually lost their self-belief and questioned their own sanity with examples given of how this led them to avoid seeking medical help. This was also found in a study of parents of children with auto-inflammatory disease where they reported doubting their own judgement in the face of medical disbelief. As in our study, this generated a persisting distrust of clinicians even after diagnosis [33].

Many patients reported that referrals and diagnosis were delayed by common misunderstandings, including that lupus can only be diagnosed in females with positive anti-dsDNA, ANA, malar rash and kidney damage. Previous research demonstrates longer delays to diagnosis reported in children, males and late-onset cases [34], and in ANA-negative patients [11]. Although SLE is more prevalent in females of child-bearing years with a Female:Male ratio of 9:1 the disease affects both genders, all ages and there are multiple other presentations, symptoms and immunological criteria for diagnosis [1, 35, 36].

The complexity and varied presentations of SLE and other related autoimmune diseases causes difficulty with the desire for clarity and certainty [37, 38]. Uncertainty and the strongly expressed desire for a clear diagnosis may be better managed by improved physician communication as to the unpredictability and evolving nature of these diseases. Lack of definitive diagnosis or delay in provision of treatment for debilitating symptoms is distressing for patients yet may have a well-considered medical reason, for example, the necessity to consider other potential causes and the balancing of risks versus benefits of medication. This forum analysis has demonstrated the great appreciation for those physicians who clearly and compassionately communicate the rationale behind these decisions and provide support with symptoms, regardless of presumed cause. Reports of dismissal and disbelief by clinicians unable to discover a cause for a new symptom were frequent, and their offers of reassurance rarely inspired confidence. Even those with long-standing disease reported misdiagnoses or no explanation—or treatment—for new, rarer [39–41] manifestations, particularly from those physicians who were felt to focus only on the more common symptoms affecting joints, skin and kidneys. Early signs of rarer manifestations were often under-investigated and undetected, until they progressed to become obvious on current (often perceived as inadequate) testing, sometimes coinciding with irreversible organ damage, including sight or hearing loss, intestinal failure and brain damage.

Improved communication and support for any co-diagnoses may also assist in acceptance and trust in cases where a co-diagnosis is accurate and helpful in terms of differentiating from active SLE for disease and symptom management. While acknowledging the very real and distressing nature of conditions with no current definitive testing, such as ME/CFS and fibromyalgia, many forum participants remained extremely sceptical as to the validity and acceptability of these as co-diagnoses in the context of autoimmune diseases, when pain, fatigue and sensory disturbance are also a regular manifestation of the primary disease.

There was a strong perception of geographical, socioeconomic, gender, age and disease inequalities of diagnosis and care, particularly regarding the Welsh gate-keeper policy. Following the BSR guidelines [1] could reduce multiple inequalities by improving knowledge and consistency in referrals, diagnosis and management. The perceived lower level of medical and social support compared to other diseases needs consideration. Although the changes to the British welfare system have adversely impacted many chronically diseased patients, the invisibility and relapsing–remitting nature of the disease, the lack of diagnostic certainty and lack of understanding regarding SLE by both physicians and welfare assessors, potentially causes greater problems for these patients accessing appropriate financial support. These differentials of power are reported in previous studies [42, 43], with difficulties accessing welfare during diagnostic journeys, potentially due to distrust in the welfare system of those lacking a definite diagnosis [42], and Whitehead and Williams hypothesising that female lupus patients struggle to be taken seriously, being ‘doubly burdened’ by gender and diagnostic uncertainty [43].

In addition to difficulties obtaining diagnosis and care, forum members also discussed the many negative consequences of the disease on their quality of life. Severe fatigue, pain and cognitive difficulties were most regularly mentioned, often causing difficulties with employment and relationships. As with other studies of chronic illness, we found that self-worth and self-identity are frequently damaged due to an inability to fulfil social [44] and caring roles.

This study has identified that the support provided by the forum, LUPUS UK and by empathetic physicians, family and friends helps mitigate invalidation and uncertainty. Clinicians providing emotional support and listening to patient’s symptoms non-judgmentally is highly valued by forum members. By presenting the patient perspective in this paper, we hope that clinicians will further consider the great impact on patients of physician communication styles and validation of patient symptoms. Irrespective of the final diagnosis, patients need to feel that their concerns are being taken seriously and that they are supported in managing their symptoms. This may mean sign posting to other services to help support the patient if the clinic lacks time/experience in the management of all symptoms, particularly those that may be multifactorial in origin.

Emotional support and the sharing of experiences is an important function of all patient groups, whether face-to-face or online and was highly valued by this group of patients. In agreement with other studies, we found that peer support helped people through interactions with others in similar situations, often by promoting self-esteem, improving self-worth [45], and reducing isolation [27]. In addition, we identified that group norms of mutual support and empathy were also found to enhance emotional resilience. Our findings were in line with Basu and Dutta’s study [46] that online communities can increase medical knowledge through health-related discussions.

Many of the LUPUS UK forum members demonstrated a high level of medical knowledge and shared up-to-date research, with multiple posts detailing how knowledge and empowerment gained through the forum assisted in diagnosis and improved care. However, the large quantity of posts requesting information on deciphering blood test results, medication enquiries, symptoms and misunderstandings of the disease, particularly among new members, may point to the need for more information and clearer explanations from clinicians, especially at diagnosis.

A strength of this study is that the forum enabled a much larger quantity of viewpoints to be analysed than traditional qualitative methods. Views stated are more likely to be free from social desirability bias as there was no researcher or physician influence on conversational direction [45]. The 24-h forum accessibility means that experiences and emotions are often reported immediately so less subject to recall bias. The limitations are that the results may not be representative of the wider SLE population due to demographic and experience bias. As with all qualitative research, the results cannot be generalised to the wider SLE population, although the large size of this group makes this less of a limitation than in traditional interview-focused research. There is limited demographic information, diagnoses are self-stated and cannot be confirmed, and disease severity, including organ involvement, is not always apparent from the conversations. Members may be those with more negative experiences who are in need of greater peer support. Negative and invalidating medical experiences may be reported more frequently than positive ones as patients seek validation from peers. In addition, patients who have had poorer explanations or persistent confusion about their disease may be more likely to post comments. While a limitation of forum analyses is the inability to request clarification on members’ experiences, we were able to reduce this limitation by discussing the research on the forum, posing questions and sharing research findings to ensure the themes identified were validated by the community.

In conclusion, whilst we cannot extrapolate these results to all SLE/systemic autoimmune patients, the overarching theme is chronic invalidation with patients who often felt poorly served—and in some cases, damaged—by health systems, with fragmented care and a lack of knowledge about the disease amongst society and many physicians. Support for the much-reduced quality of life could be improved by more access to specialist support (e.g. specialist nurses, physiotherapy and psychosocial support), signposting of all patients towards relevant charities, and developing additional methods of self-management education and peer support. Improved support for all patients with initially medically unexplained symptoms is an urgent requirement; not only to avoid damage from psychogenic misdiagnoses, but also because a medical—and potentially treatable—explanation was usually subsequently discovered in these patients after more detailed testing or consultation with appropriate specialists. This study highlights the importance of earlier diagnosis, cross-specialism support and empathetic communication. In addition, there is a need for more appropriate and prompt investigations in those diagnosed, as not all new symptoms should be automatically attributed to the primary autoimmune disease yet ANY new sign or symptom could be related to systemic autoimmunity.

## Electronic supplementary material

Below is the link to the electronic supplementary material.

Supplementary file1 (DOCX 18 kb)
