# Supplementary material for: ‘But you don’t look sick’: a qualitative analysis of the LUPUS UK online forum
Source: Rheumatol Int. 2020 Oct 26;41(4):721–32. doi: 10.1007/s00296-020-04726-x (PMC7952333; doi:10.1007/s00296-020-04726-x)
Supplement: Supplementary file 1 — Supplementary file1 (DOCX 18 kb) [file 296_2020_4726_MOESM1_ESM.docx]

Supplementary Information 1

**Supplementary Information 1: Details of methods**

**Research ethos and Involving the community**

The ethical principles underlying this study were primarily to give these forum members a stronger voice in medical literature and further their interests [47], whilst protecting the community and individuals from harm. Our ethos was that this research would be carried out by and with the community [48], rather than on the community. To achieve this aim, several long-standing members of the group were approached by messaging from the lead researcher (MS).

MS’s membership of the forum as a patient with SLE, and discussions with many other patients and LUPUS UK, generated the idea for this research, feeling that it would be very beneficial to patients if policy makers, researchers and clinicians could better understand – from the patient perspective – some of the many challenges faced by this group. Members were selected to be offered membership of the study team from the nature of their posts and responses. Selection criteria were: to be well-respected members, demonstrating excellent knowledge and sharing this with other members, providing compassionate, supportive responses and demonstrating an objectivity in their views that is essential for research of this nature. Multiple members were found to fit these criteria and two initially joined the research team (MAB and CB) followed by MB and LH. Two of the patient team members are also ex-clinicians.

**Ethical considerations**

Forum analyses raises a number of unique ethical issues with no definitive guidance [49], and so different research groups approach the ethical concerns in different ways. Our primary consideration was to ensure anonymity and not disrupt the usual group dynamics and communication. Feeling observed for research purposes from external sources may alter the open nature of the group and cause discomfort in some communities. It was important that the individuals and group as a whole do not perceive the analysis and publishing of common themes as a violation of their privacy. These members are aware that the forum is in the public domain and users have signed an agreement on joining that the forum may be viewed and analysed by HealthUnlocked (the owners of the forum) and researchers. Passive analysis is regularly occurring on many of these forums without the members’ knowledge yet it is possible that many do not consider that the posted material will be used for research purposes. We therefore fully informed the forum members of the rationale and purpose of the study and give members an option to opt out of any of their posts/responses being used in the analysis.

As forums are a relatively novel method of data collection, we utilised an additional ‘microethics’ adaptive approach where we continually reviewed and were vigilant for any arising potential ethical issues.

Involving the community in the research was a priority. The research was discussed with multiple forum members and an information sheet was posted on the forum detailing the research and how permission for quotes would be obtained.

We obtained written consent from any participant where we wished to use a direct quote. Permission was sought by LUPUS UK (CW) with only two forum members refusing permission to be quoted. We protected both real world and forum identities by quotes being anonymised and only attributed to gender, decade of age and country.

**Research team and mitigating bias**

The wider research team was selected to incorporate a wide range of relevant experiences, perspectives and skills and included: rheumatologists, SLE specialists, patients, behavioural scientists, and LUPUS UK staff. This multidisciplinary team, and an awareness and discussions of potential bias in both patient and physician team members, mitigated any bias arising from past individual experiences.

Although all of the patient co-authors posted regularly, both before and after the study commenced, they did so as fellow patients rather than researchers, continuing their interactions as normal. However, in order to ensure that discussions on emerging themes between the co-authors during the lengthy immersion period did not lead to posts that could potentially bias the findings, no data or quotes were used from patient co-author posts from the date that co-author joined the study.

**Qualitative analysis**

MS, MAB, CB, MB and LH all continued being immersed in the forum as patients, with all viewing posts/responses most days and contributing as normal, health depending. In addition, they had an email group with regular (on average every 3-4 days) discussions about the common experiences on the forum, with ideas as to the main categories to analyse and report emerging from these discussions. The forum moderators, PH and CW, shared their views from many years (11 years combined) of moderating the forum (daily), and the SLE specialists, DD’C and CG, clarified whether arising themes were in line with physician experiences of this patient group.

A further period of more structured analysis followed using thematic analysis, from a coding frame developed from the categories arising from the discussions following immersion in the forum, with a selection of posts initially double coded by PH and CW in order to ensure agreement and consistency of the coding frame. The coding frame and initial categories were shared with the wider team.

Thematic analysis was selected as the methodological approach. Forum conversations were coded by MS and analysed in reverse chronological order until it was felt that a reasonable degree of theoretical saturation had been achieved (Oct 2019-Mar 2019), followed by a further review of selected relevant historical posts as far back as Sep 2012, searched for from key words (and their opposites) from the emerging themes (e.g. belief/disbelief, mild/severe lupus). Theoretical saturation was felt to be achieved when (1) the themes together addressed all the main research questions; (2) the themes provided adequate explanations of corresponding phenomena when applied to additional forum conversations; and (3) additional forum posts did not provide new relevant insights.

Coding and initial analysis using thematic analysis [20] occurred after every few days of posts. The stages of analysis involved:

1) Immersion in the forum – Full Immersion in current and historical posts was the initial stage of analysis and continued throughout the 2 years of the study.

2) Developing and agreeing an initial coding (classification) scheme – The coding scheme was agreed by co-authors and tested by MS, PH and CW. Codes were generated from the forum posts, for example ‘misdiagnosis’ ‘test result queries’ ‘positive physician interactions’

3) Coding the data – Forum posts and responses were then coded by MS, using NVivo 11 software

4) Refining and re-coding – Several categories of post did not fully fit the initial coding frame, so additional codes were added after discussion with the team, and several codes were combined. Coding continued until saturation point was reached where no new novel insights were forthcoming.

5) Identifying commonly occurring themes - Codes were then combined into broader categories with the emergence of key themes becoming apparent from both observing the uncoded conversations, the coded data, and in-depth discussions concerning the data from the team.

**Community approval and presentation of results**

Common themes and concepts were then discussed and agreed with CG, DDC, FN and SS and multiple patients. This ensured patient, rheumatology and psychology expert perspectives were utilised in analysis, and consistency between the findings and data presented.

Quotes were found on the forum that illustrated the key findings. Clarity of presentation of themes was confirmed by patients and rheumatologists who reviewed the manuscript.

Member checking [50] was an important component of ensuring accurate representation of the overall community ‘voice’ The draft results and discussion sections of the paper were posted on the forum by PH and members were asked for their comments and whether they agreed/disagreed with the findings. An open discussion was had on the forum and MS responded to queries and suggestions, re-drafting the paper where necessary. The forum members views were also ascertained by several questions being asked by PH and responses followed up MS regarding initial themes and priorities generated from both our survey paper [11] and the initial immersion period in the forum.

Supplementary Information References;

47. Christians CG (2003) Ethics and politics in qualitative research. In: Denzin NK, Lincoln, YS, ed. The Landscape of Qualitative Research - theories and issues. London: Sage Publications, 2003: 208-243.

48. Human Inquiry: A Sourcebook of New Paradigm Research. Edited by Peter Reason and John Rowan. New York: John Wiley & Sons, (1981)

49. Harriman S, Patel J (2014)The ethics and editorial challenges of internet-based research. BMC medicine, 12:124

50. Cho J and Trent A (2006) validity in qualitative research. Qualitative research, 6(3), 319-340
